# Supplementary material for: The Transcriptional Landscape of Marek’s Disease Virus in Primary Chicken B Cells Reveals Novel Splice Variants and Genes
Source: Viruses. 2019 Mar 16;11(3):264. doi: 10.3390/v11030264 (PMC6466439; doi:10.3390/v11030264)
Supplement: Supplementary file 1 [file viruses-11-00264-s001.zip › Supplementary_Material.pdf]

viruses

MDPI

Supplementary Material for:

The Transcriptional Landscape of Marek's Disease Virus in Primary Chicken B Cells Reveals Novel Splice Variants and Genes.

by L. Bertzbach and F. Pfaff *et al.*

Supplementary Methods

Method S1: Generation and *In Vitro* Characterization of RB1BΔMetICP0

To generate an ICP0 knockout mutant and a virus with a tagged ICP0, we mutated the only start codon ATG to a double stop codon (TTGTTG) in the RB-1B strain (RB1B_ΔMetICP0) and created a virus harboring an HA-tagged ICP0 (RB1B_ICP0-HA) respectively, using two-step Red-mediated mutagenesis. Final clones were confirmed by multiple restriction fragment length polymorphism (RFLP) analyses, PCR, and Sanger sequencing of the target region. The viruses were reconstituted and propagated on CEC for up to six passages, and infected cells were stored in liquid nitrogen until further use. All virus stocks were titrated on fresh CEC. The RB1B_ΔMetICP0 replication was analyzed using plaque size assays. One million CECs were infected with 100 PFU and fixed at 6 days post-infection. Fixed cells were stained with an anti-MDV chicken serum, and plaques were visualized using an anti-chicken Alexa Fluor 488 secondary antibody. Images of 50 randomly selected plaques were taken, and plaque diameters were determined using the Image J Software (NIH). For western blot analysis, cells were harvested and lysed in radioimmunoprecipitation assay buffer (RIPA). Lysates were mixed with SDS sample loading buffer and denatured for 5 min at 95°C prior to SDS-PAGE separation. Proteins were transferred onto a polyvinylidene difluoride (PVDF) membrane (Carl Roth; Karlsruhe, Germany) using the BioRad wet blot system (BioRad Laboratories; München, Germany) for 1 h at 9 V. Membranes were blocked and incubated for 16 hours at 4°C with an HA-Tag (6E2) mouse monoclonal antibody (1:1000). Proteins were visualized using a horseradish peroxidase-conjugated anti-mouse secondary antibody and membranes were stained with a western blot detection reagent (enhanced chemiluminescence, ECL-prime, GE Healthcare; Chicago, IL, USA). The signal was recorded with the Chemi-Smart 5100 detection system (Peqlab; Erlangen, Germany).

Supplementary Figures

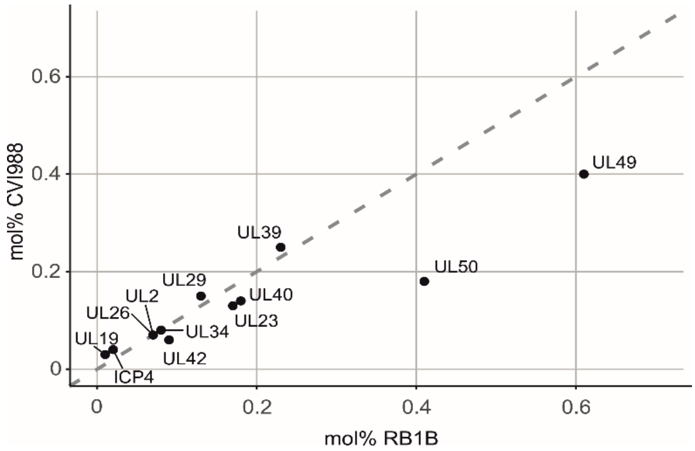

Figure S1: Protein concentration (mol%) scatterplot comparing levels of detected viral proteins in RB1B and CVI988 infected primary chicken B cells. The dashed lines indicate equal distribution.

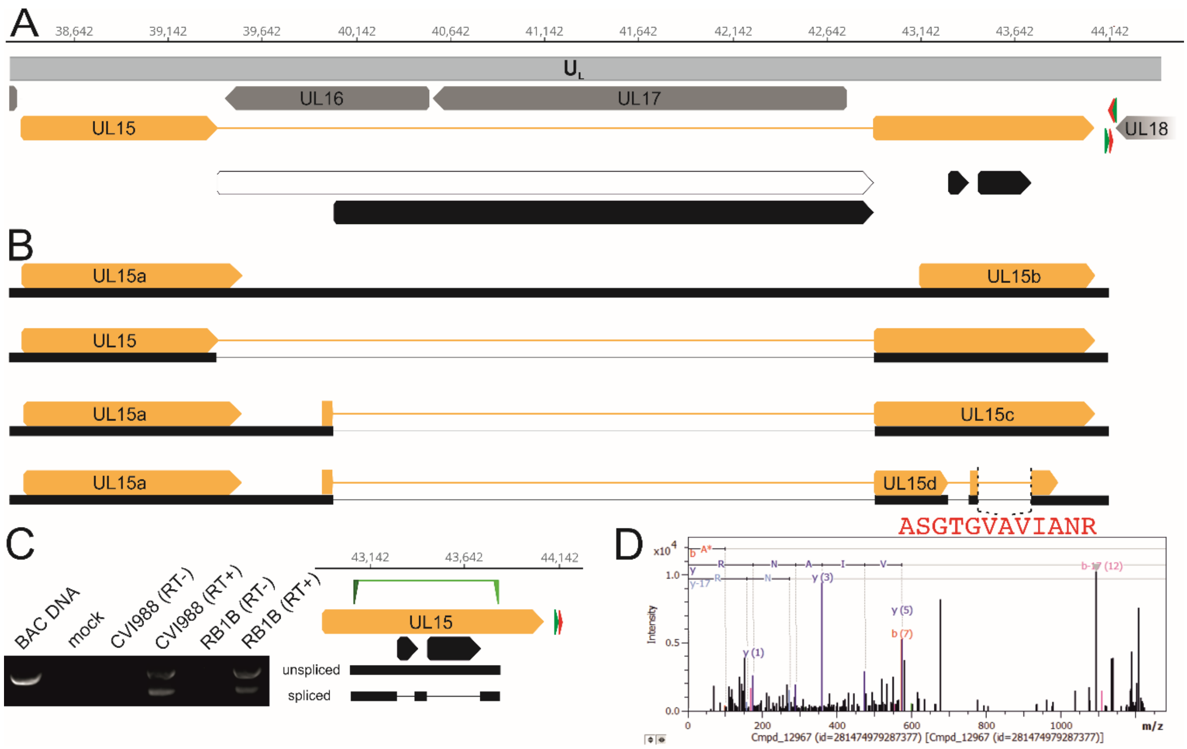

Figure S2: **Detected splice variants of UL15. (A)** UL15 is currently annotated as spliced gene (orange arrows) in MDV Refseq NC002229 (positions) with an intron (white arrow) that spans the CDS of UL16 and UL17 (grey arrows). In addition to this canonical splice event, we detected at least three more possible introns (black arrows). Detected poly(A) cleavage cluster and the correlating motif are highlighted as red and green arrows, respectively. **(B)** The black bars indicate possible UL15 transcripts, while the orange arrows indicate possible open reading frames. Splicing results in at least five possible proteins: UL15, UL15a, UL15b, UL15c and UL15d. **(C)** RT-PCR was performed to validate the splicing event that results in UL15d. PCR products (black bars) were derived using forward/reverse primers (green markers) to amplify the respective intron-flanking regions for UL15d. The representative gel images illustrate the simultaneous presence of spliced and unspliced RNA of RB1B and CVI988. **(D)** The exon-exon junction within UL15d matched a peptide (red) found by MALDI-TOF/TOF. Only y- and b- series are annotated.

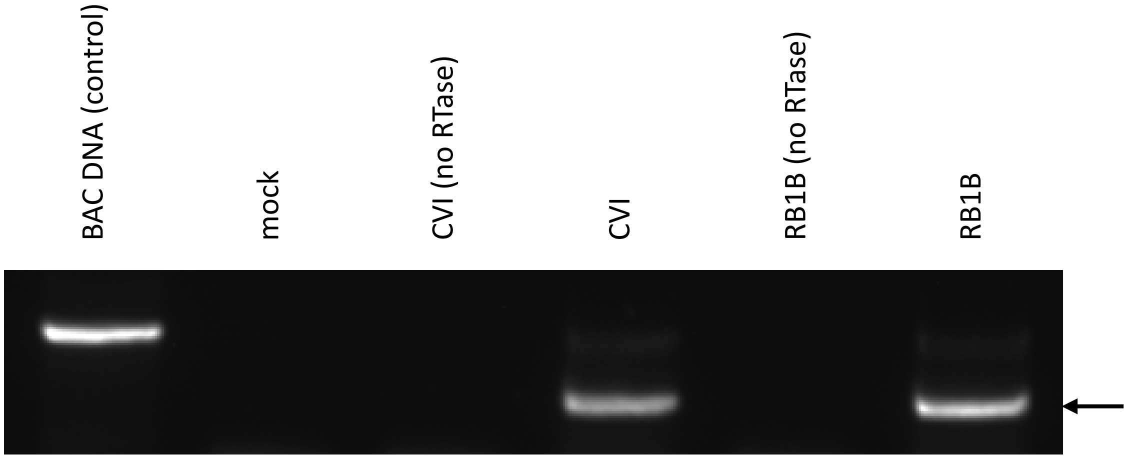

Figure S3: **RT-PCR confirmation of splicing events in pp38.** PCR products were derived using forward/reverse primers to amplify the respective intron-flanking regions for pp38. The representative gel images illustrate the results of RT-PCR analysis. The black arrow indicates the spliced form.

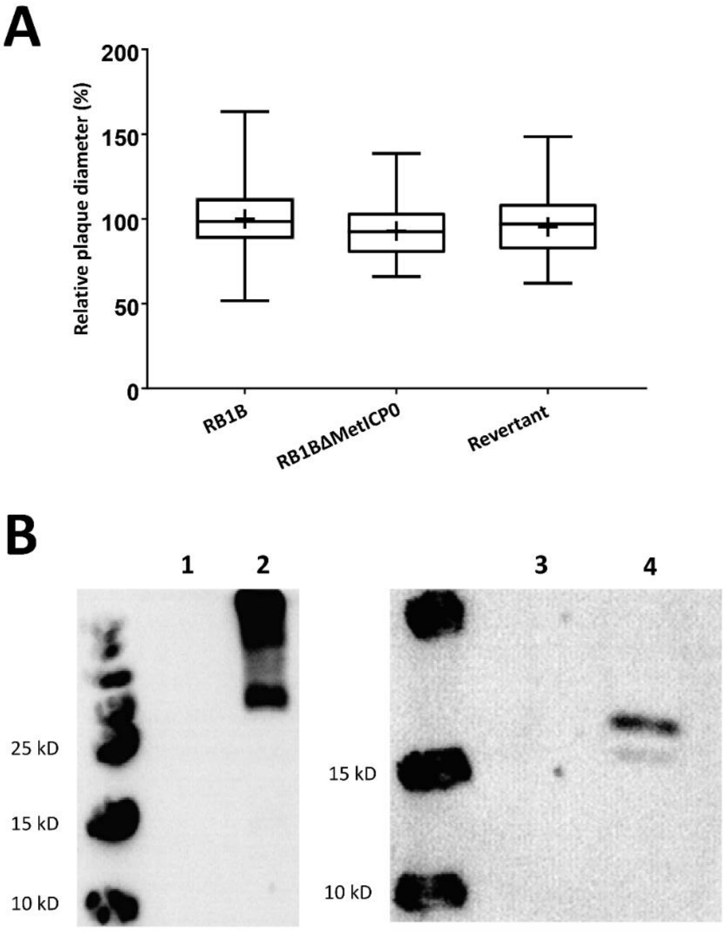

Figure S4: **Plaques size assays and western blot analysis for the ICP0 mutant RB1BΔMetICP0.** *In vitro* characterization of the RB1BΔMetICP0 mutant confirmed that it is dispensable for virus growth and cell-to-cell spread. **(A)** Relative plaque diameters of 50 randomly selected plaques per virus are shown as box plots with 95% confidence intervals and SD. **(B)** Western blot analysis detecting an RB1B virus mutant harboring an HA-tagged ICP0 (RB1B_ICP0-HA) (1), an HA tag positive control (2), and RB1B_ICP0-HA (3) compared to a pVitro-plasmid-expressed, HA-tagged ICP0 (4).

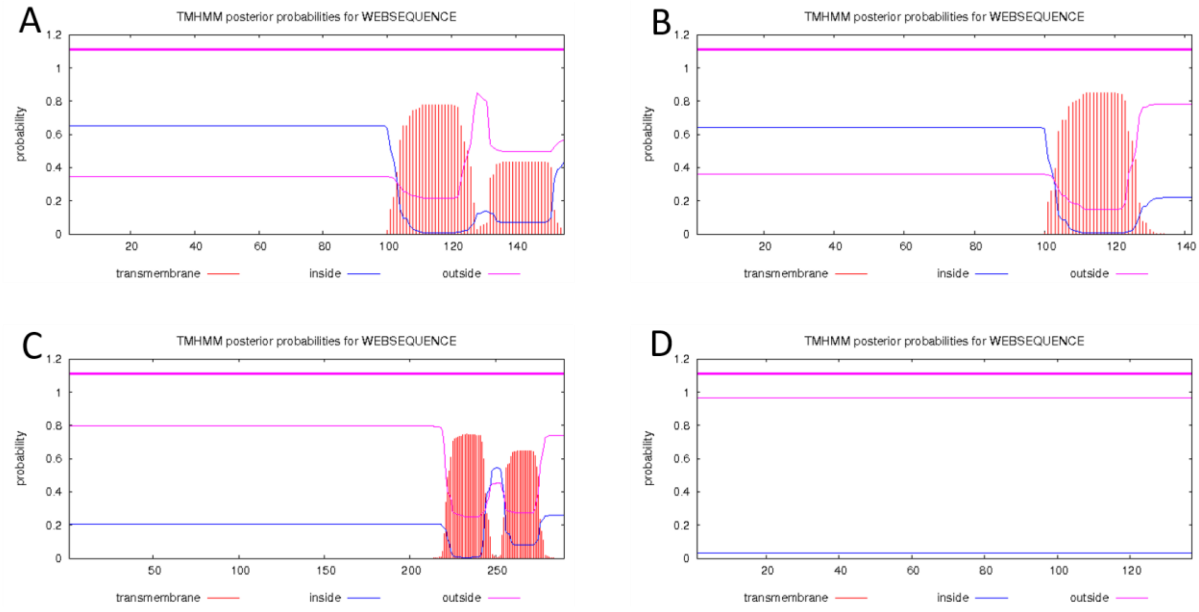

Figure S5: Transmembrane helix prediction for spliced and unspliced pp24 (A and B) and pp38 (C and D). Protein with two transmembrane helices (unspliced forms, A and C) and with only one helix (pp24 spliced, B) and no transmembrane helix (pp38 spliced, D). Prediction was done using the TMHMM Server v. 2.0.

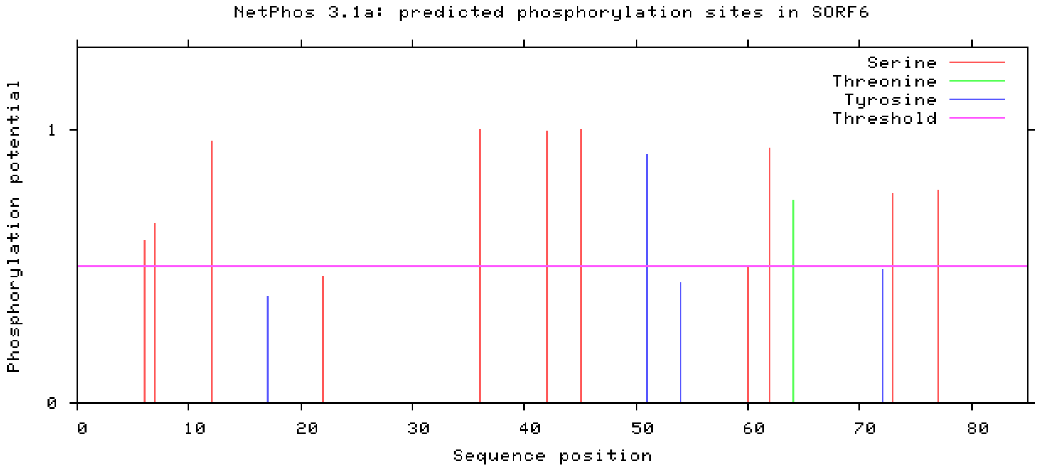

Figure S6: Prediction of serine, threonine or tyrosine phosphorylation sites in the hypothetical MDV protein SORF6 encoded on the Us segment. Prediction was done using the NetPhos 3.1 Server.

Supplementary Tables

Table S1: Marek's disease virus (MDV) proteins detected by mass spectrometry

ORF	Gene	Protein Description
MDV014	UL2	Uracil-DNA glycosylase
MDV031	UL19	Major capsid protein
MDV036	UL23	Thymidine kinase
MDV038	UL26	Capsid scaffolding protein
MDV042	UL29	Major ssDNA binding protein
MDV047	UL34	Nuclear egress protein 2
MDV052	UL39	Ribonucleoside-diphosphate reductase large subunit
MDV053	UL40	Ribonucleoside-diphosphate reductase small subunit
MDV055	UL42	DNA polymerase processivity subunit
MDV062	UL49	Tegument protein VP22
MDV063	UL50	Deoxyuridine 5'-triphosphate nucleotidohydrolase (DUT)
MDV084/MDV100	SORF1	Major viral transcription factor ICP4

Table S2: Primers used in this study

Gene	Direction	Sequence
MDV010 (vLIP)	For	GATCCATGGAACTGCATTGG
	Rev	TGGGATTCCCATGAAAGGTA
MDV012 (ORF012)	For	CTTACTCTCGCGCCTGGTAA
	Rev	GGGGATCAGGCTCCTTAGAA
MDV057 (gC)	For	TGCATACCCACGTCAATCTG
	Rev	TTTCTATGGCGATCGATGGT
MDV027 (UL15)	For	AAGGATCGGCTGATGATCTT
	Rev	CCGAATTAAACTGGGCGATA
MDV073 (pp38)	For	AATTCGCTTAATCTCCGCCT
	Rev	GGAATTCGAAGCAGAACACG
novel gene in US (SORF6)	For	TGGTTTATCGATTTCCGGAA
	Rev	TTCGACGAATTAAGCTCGAT

Table S3: Summary of RNA-seq read mapping

Library	Virus	Reads	Average read length	Uniquely mapped reads	Uniquely mapped reads %
MDV-CVI988_1_lib02435	CVI988	15.585.559	141	2.020.992	12.97
MDV-CVI988_2_lib02436	CVI988	15.662.671	135	1.076.218	6.87
MDV-CVI988_3_lib02437	CVI988	16.987.196	136	1.977.331	11.64
MDV-RB1B_1_lib02432	RB1B	17.798.972	133	2.095.271	11.77
MDV-RB1B_2_lib02433	RB1B	16.582.704	111	1.760.125	10.61

Table S4: Introns identified from RNA-seq data.

Intron start	Intron stop	Orientation	Read number	Frequency in CVI988	Frequency in RB1B	Intron length
14218	14477	+	472	0.028	0.010	260
14631	14700	+	1062	0.029	0.072	70
17540	17621	+	7734	0.945	0.883	82
23570	25010	+	387	0.938	0.814	1441
37541	39215	+	67	1.000	0.000	1675
39404	42884	+	33	0.149	0.000	3481
40023	42884	+	146	0.000	0.658	2862
42651	42884	+	42	0.977	0.000	234
43282	43389	+	409	0.022	0.598	108
43444	43723	+	646	0.071	0.422	280
43444	48493	+	35	0.033	0.000	5050
44447	46036	+	115	0.504	0.000	1590
46711	48559	+	61	0.310	0.000	1849
47645	48559	+	23	0.404	0.000	915
47909	48559	+	20	0.452	1.000	651
48026	48551	-	170	0.039	0.311	526
48633	48904	+	264	0.632	0.031	272
49048	50816	+	276	0.412	0.000	1769
56222	57133	+	237	0.514	0.000	912
65264	65913	+	82	0.000	0.953	650
65591	65913	+	11	0.268	0.000	323
95701	102920	-	51	0.221	0.000	7220
103244	103388	+	2375	0.224	0.082	145
103285	103388	+	8900	0.820	0.628	104
103542	104450	-	16	0.615	0.000	909
111959	112359	-	368	0.447	0.000	401
123201	123695	-	23	0.043	0.023	495
123310	123803	-	48	0.096	0.000	494
125947	126222	+	24	0.889	0.000	276
126269	127407	-	20	0.002	0.000	1139
126300	126729	+	24	0.960	0.000	430
126904	127407	-	3695	0.214	0.172	504
126904	127437	-	181	0.006	0.011	534
127855	128016	-	25341	0.573	0.660	162
127917	128016	-	4070	0.102	0.080	100
127939	128016	-	2843	0.073	0.052	78
128559	129719	-	88	0.044	0.000	1161
128572	130900	+	5725	0.046	0.269	2329
128764	130900	+	4670	0.031	0.036	2137
129809	130865	-	1608	0.833	0.557	1057
129853	130865	-	130	0.075	0.000	1013
129866	130900	+	3475	0.022	0.021	1035
130958	131423	-	2235	0.564	0.290	466
131633	131762	-	784	0.586	0.000	130
131683	131762	-	177	0.132	0.000	80
132126	133050	-	26	0.121	0.579	925
132975	133061	+	59	0.967	0.000	87
135044	135780	-	282	0.000	0.310	737
135161	135780	-	414	0.720	0.040	620
135943	136062	-	287	0.571	0.531	120
138129	138303	+	23824	0.947	0.916	175
138440	138537	+	69973	0.913	0.941	98
138440	138521	+	1732	0.027	0.014	82
143491	152141	-	86	0.025	0.025	8651
144052	144604	+	118	0.000	0.423	553
144052	144376	+	77	0.182	0.254	325
144052	144180	+	47	0.000	0.168	129
144494	144604	+	497	0.000	0.823	111

144724	144996	+	48	0.960	0.000	273
145239	147059	+	37	0.213	0.011	1821
147123	147209	+	401	0.316	0.360	87
147608	147678	+	1074	0.609	0.467	71
147608	147819	+	486	0.000	0.254	212
147787	148156	+	1744	0.832	0.819	370
148246	148330	+	692	0.477	0.624	85
148407	150128	+	457	0.138	0.221	1722
148407	149466	+	206	0.000	0.122	1060
150744	152141	-	442	0.135	0.098	1398
150945	152141	-	1684	0.443	0.644	1197
152280	152624	+	24	0.012	0.000	345
164391	164465	-	1824	0.706	0.434	75

Table S5: Poly(A) cleavage sites, polyadenylation signals and polycistronic transcripts identified in MDV transcriptomes.

Position of poly(A) cleavage site	Orientation	Read coverage	Position of associated motif	Motif sequence	Distance	Associated transcripts
15163-15169	+	21	15141-15146	ATTAAA	16	MDV008
15285-15292	+	176	15268-15273	AATAAA	11	MDV008
16898-16910	+	219	16887-16892	AATAAA	5	MDV010
16901-16901	-	7	16919-16924	AATAAA	17	unknown
19058-19068	+	377	19041-19046	AATAAA	11	MDV012
19668-19668	-	8	n.d.			unknown
21620-21620	-	4	21642-21647	AATAAA	21	MDV016, MDV017
21620-21632	+	109	21603-21608	AATAAA	11	MDV013, MDV014, MDV015, MDV015.5
21726-21732	-	8	21748-21753	AATAAA	15	MDV016, MDV017
21726-21738	+	44	21709-21714	AATAAA	11	MDV013, MDV014, MDV015, MDV015.5
28246-28257	-	5	28272-28277	AATAAA	14	MDV020
28256-28266	+	38	28237-28242	AATAAA	13	MDV018, MDV019
30453-30454	-	8	30471-30476	AATAAA	16	MDV021
31641-31641	-	11	n.d.			unknown
32127-32127	-	13	32147-32152	AATAGA	19	unknown
34530-34534	-	47	34550-34555	AATAAA	15	MDV023, MDV024, MDV025
34532-34535	+	53	34512-34517	AATAAA	14	MDV022
36883-36889	-	36	36913-36918	ATTAAA	23	MDV026, MDV028, MDV029
37074-37074	-	4	n.d.			MDV026, MDV028, MDV029
37264-37267	-	8	37282-37287	AATAAA	14	MDV026, MDV028, MDV029
44138-44162	-	274	44168-44173	AATAAA	5	MDV030, MDV031, MDV032
44144-44150	+	136	44123-44128	AATAAA	15	MDV027
52458-52461	-	11	52479-52484	AATAAA	17	MDV034, MDV036
52460-52465	+	13	52441-52446	AATAAA	13	MDV033
59083-59085	+	7	59064-59069	AATAAA	13	MDV035, MDV037
61008-61011	-	208	61025-61030	AATAAA	13	MDV040, MDV041
61009-61009	+	188	60991-60996	AATAAA	12	MDV038, MDV039
64872-64872	-	3	64889-64894	AATAAA	16	unknown
66217-66231	-	97	66243-66248	AATAAA	11	MDV042
69388-69392	-	55	n.d.			unknown
73618-73621	-	86	73636-73641	AATAAA	14	MDV044, MDV046
73842-73862	+	101	73822-73827	AATAAA	14	MDV043
78413-78419	+	20	78394-78399	AATAAA	13	MDV045, MDV047, MDV048
78421-78422	-	9	78438-78443	AATAAA	15	MDV049
79493-79493	+	5	79469-79474	ATTAAA	18	unknown

85728-85732	-	6	85749-85754	AATAAA	16	MDV050
97360-97362	-	10	97379-97384	ATTAAA	16	MDV054
97438-97442	+	50	97421-97426	AATAAA	11	MDV051, MDV052, MDV053
100955-100955	+	6	100920-100925	AAAAAA	29	MDV055
102019-102024	+	167	102001-102006	AATAAA	12	MDV056
103709-103722	+	7	103695-103700	ATTAAA	8	MDV057
105182-105190	-	78	105201-105206	AATAAA	10	MDV059, MDV060
105185-105185	+	11	105175-105180	AATAAA	4	MDV058
109707-109710	-	167	109725-109730	AATAAA	14	MDV061, MDV062, MDV064
113737-113758	-	78	n.d.			MDV065
113742-113747	+	288	113723-113728	AATAAA	13	MDV063
120437-120464	+	139	120422-120427	AATAAA	9	MDV066, MDV067, MDV068
120451-120459	-	46	120472-120477	AATAAA	12	MDV069
121971-121985	+	124	121957-121962	AATAAA	8	MDV070
121975-121980	-	6	121999-122004	ATTAAA	18	MDV071, MDV072
122824-122824	+	29	n.d.			unknown
126400-126411	-	487	126421-126426	AATAAA	9	MDV072.5, MDV073
128589-128619	+	48	128574-128579	AATAAA	9	RLORF12
128620-128637	+	334	128601-128606	AATAAA	13	RLORF12
128844-128863	+	59	n.d.			ORF3
128901-128931	+	72	n.d.			ORF3
128934-128957	+	167	128907-128912	ATTAAA	21	ORF3
128975-128992	+	7	n.d.			ORF3
129287-129304	+	83	129276-129281	ATTAAA	5	unknown
129500-129503	+	7	n.d.			unknown
129633-129633	-	8	129655-129660	AATAAA	21	RLORF11
130233-130238	+	1725	130217-130222	AATAAA	10	unknown
132160-132173	+	298	132143-132148	ATTAAA	11	14 kDa lytic phase protein, RLORF9
136325-136329	-	11	n.d.			unknown
137292-137296	+	9	137271-137276	ATTAAA	15	L1
138580-138584	-	6	138599-138604	AATAAA	14	unknown hypotetical protein
138782-138788	+	16	138762-138767	ATTAAA	14	MDV078
139043-139049	+	28	139025-139030	ATTAAA	12	unknown hypotetical protein
142968-142977	-	18	142994-142999	AATAAA	16	MDV082, MDV084
143058-143068	-	27	143083-143088	AATAAA	14	MDV082, MDV084
145732-145732	-	3	n.d.			unknown
148941-148941	-	3	n.d.			unknown
153357-153360	-	6	153384-153389	AATAAA	23	unknown
155661-155669	-	3	155686-155691	AATAAA	16	MDV090, MDV091
155696-155706	+	80	155678-155683	AATAAA	12	MDV087, MDV088, MDV089
163958-163964	-	7	n.d.			unknown
163959-163968	+	189	163941-163946	AATAAA	12	MDV092, MDV093, MDV094,MDV095, MDV096
164076-164096	-	97	164115-164120	AATAAA	18	SORF6, MDV097

n.d.: No canonical motif detected.
